# Association of 206 Brain Structural Connectivity with Different Types of Strokes: A Mendelian Randomization Study

**DOI:** 10.1523/ENEURO.0209-25.2025

**Published:** 2025-10-10

**Authors:** Xinwei Wang, Yongchun Peng, Yimeng Gao, Wenjin Zhou, Tao Huang, Zizhuang Peng

**Affiliations:** ^1^The Second Clinical College of Guangzhou University of Chinese Medicine, Guangzhou 510000, China; ^2^Department of Neurosurgery, Guangdong Provincial Hospital of Chinese Medicine, Guangzhou 510000, China

**Keywords:** Mendelian randomization analysis, relationship, stroke, structural connectivity, white matter

## Abstract

The association between brain structural connectivity (BSC) and different subtypes of stroke has not been reported. The current study determined whether some BSC patterns may contribute to the risk of stroke. A two-sample, bidirectional, multivariate Mendelian randomization (MR) analysis was performed. Genome-wide association summary statistics for BSC were obtained from the GWAS Catalog at the European Bioinformatics Institute, while stroke outcome data were obtained from the FinnGen study for intracerebral hemorrhage (ICH) and from the MEGASTROKE Consortium for ischemic stroke (IS) and its subtypes. A colocalization analysis was performed to determine whether the association between BSC and stroke was driven by loci within genomic regions. Reverse MR was performed to evaluate potential stroke-induced changes in BSC. Among the significant findings, left hemisphere (LH) somatomotor network-to-LH salience/ventral attention network white matter (WM) structural connectivity (SC) [OR = 1.30; *p* = 5.96 × 10^−4^; *p* value after Bonferroni’s correction [*p.bfr*] = 0.0125] and right hemisphere (RH) dorsal attention network (DAN)-to-thalamus WM-SC (OR = 1.23; *p* = 1.60 × 10^−3^; *p.bfr* = 0.0125) were shown to have a positive association with the risk of IS. RH DAN-to-amygdala WM-SC (OR = 0.78; *p* = 1.26 × 10^−3^; *p.bfr* = 0.0125) showed a negative relationship with the risk of IS. A high LH somatomotor network-to-RH visual network WM-SC (OR = 1.62; *p* = 9.10 × 10^−3^; *p.bfr* = 0.025) was associated with an increased risk of large atherosclerotic stroke. In conclusion, the results of the current study provided some evidence from the perspective of genetics that different BSCs may have close associations with ICH, IS, and stroke subtypes. These findings may facilitate the screening and the risk stratification for stroke patients.

## Significance Statement

This study provided some evidence from the perspective of genetics that the variation in structural connectivity between some brain regions (BSC) may closely relate to differential risk of stroke subtypes. The findings suggest BSC can be used as an early risk marker for the screening of stroke patients. Further investigations of the underlying cerebrovascular and neurophysiologic mechanisms are still needed for the close association between BSC and stroke.

## Introduction

Stroke is considered the second leading cause of deaths worldwide (GBD 2019 Stroke Collaborators, 2021). According to a report published by the Lancet Neurology Commission, a 50% increase in stroke mortality is expected by 2050 compared with 2020 (Feigin and Owolabi, 2023). Stroke often occurs before the onset of clinical symptoms. Although the structural damage associated with stroke lesions is focal, the impact of this structural damage on brain function may be localized but also affects brain networks. Distant dysfunction may also occur in areas relative to the stroke-associated lesion (Sotelo et al., 2020).

White matter (WM) is almost entirely composed of myelinated axon bundles. WM fiber bundles form the structural basis of large-scale brain networks (Sporns et al., 2005). The interactions between brain regions can be measured directly with functional connectivity MRI (Messaritaki et al., 2021; Lv et al., 2024), which provides a considerable view into different functional systems throughout the brain. “Connectome” was coined by Sporns et al. (2005) and refers to the complete set of structural connections between neurons in the brain. Brain structural connectivity (BSC) has significant genetic associations with various neuropsychiatric and cognitive traits, suggesting that alterations in BSC often affect brain health and cognitive function (Zhao et al., 2021; Sha et al., 2023; Wainberg et al., 2024).

An association between BSC and stroke has been reported but has not been separated into stroke subtypes and BSC (Sotelo et al., 2020; Wang et al., 2023). Mendelian randomization (MR) has emerged in recent years by selecting expose-associated single-nucleotide polymorphisms (SNPs) as instrumental variables (IVs). MR evaluates the causal effects of exposure to genetic proxies on outcomes (Richmond and Davey Smith, 2022). Specifically, IVs alternatively mimic randomized controlled trials (RCTs), which largely avoid the effect of confounding factors in biasing causal effects (Burgess et al., 2013).

Therefore, a two-way MR analysis was performed to comprehensively explore the potential relationship between 206 types of BSCs and multiple types of strokes using pooled data from a genome-wide association study (GWAS).

## Materials and Methods

### Study design

**Figure 1.**
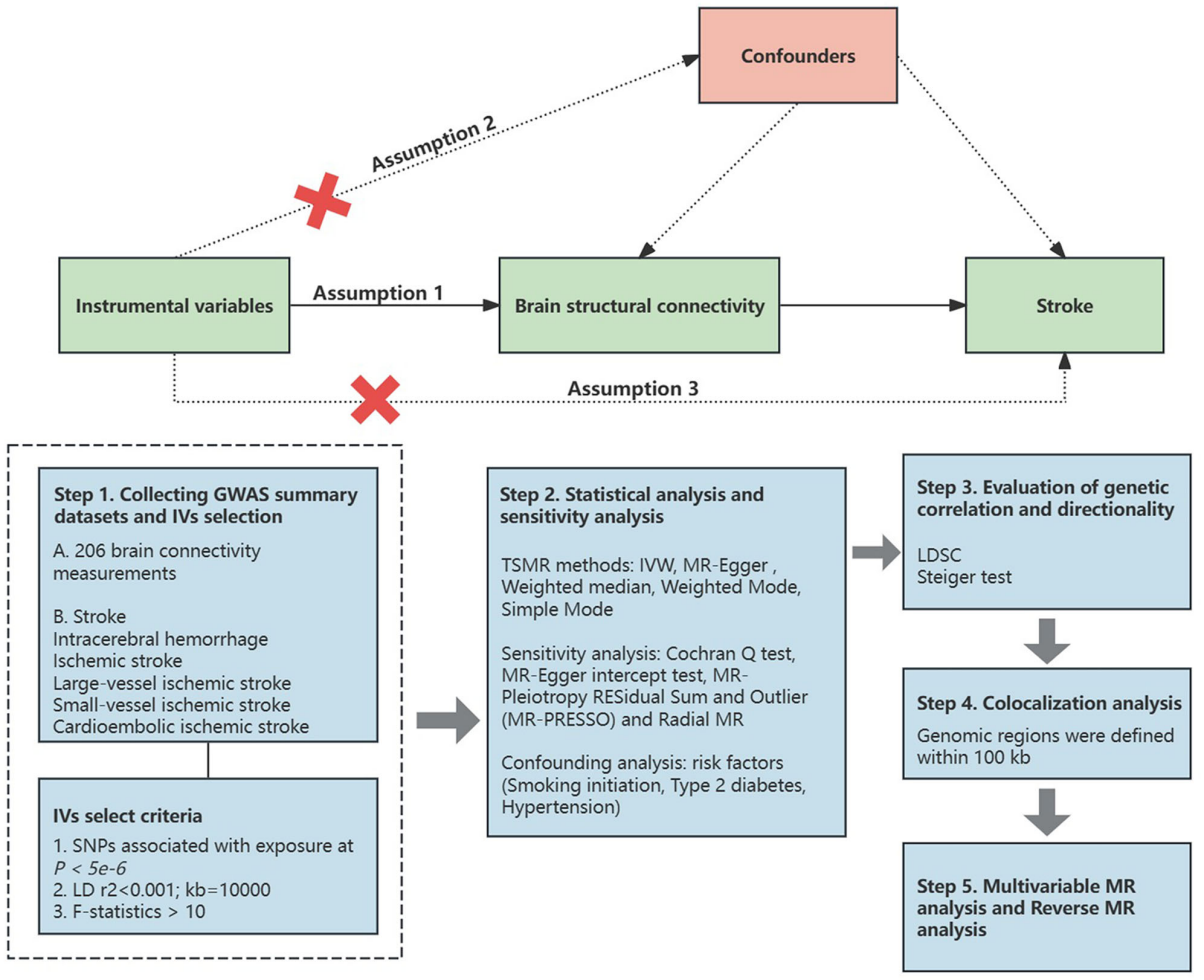
The MR analysis flowchart. Hypothesis 1, IVs closely related to the exposure of interest; Hypothesis 2, IVs not related to confounding factors; Hypothesis 3, IVs that has nothing to do with the outcome and only affects the outcome through exposure. SNPs, single-nucleotide polymorphisms; LD, linkage disequilibrium; IVW, inverse-variance weighted; LDSC, linkage disequilibrium score; MR-PRESSO, MR-pleiotropy residual sum and outlier.

An effective MR study should conform to the following three hypotheses: (1) an IV is closely related to the exposure of interest; (2) an IV is not associated with confounding factors; and (3) an IV is not associated with the results and only affects the results through exposure (Boef et al., 2015). All MR analyses were performed in the R software (4.4.0) through packages, including two-sample MR, MR-pleiotropy residual sum and outlier (MR-PRESSO), radial MR, coloc, and MR. All MR analyses followed the guidelines outlined in the STROBE-MR statement (Burgess et al., 2019). Figure 1 shows a summary of the study.

### GWAS data for 206 BSC and stroke

**Figure 2.**
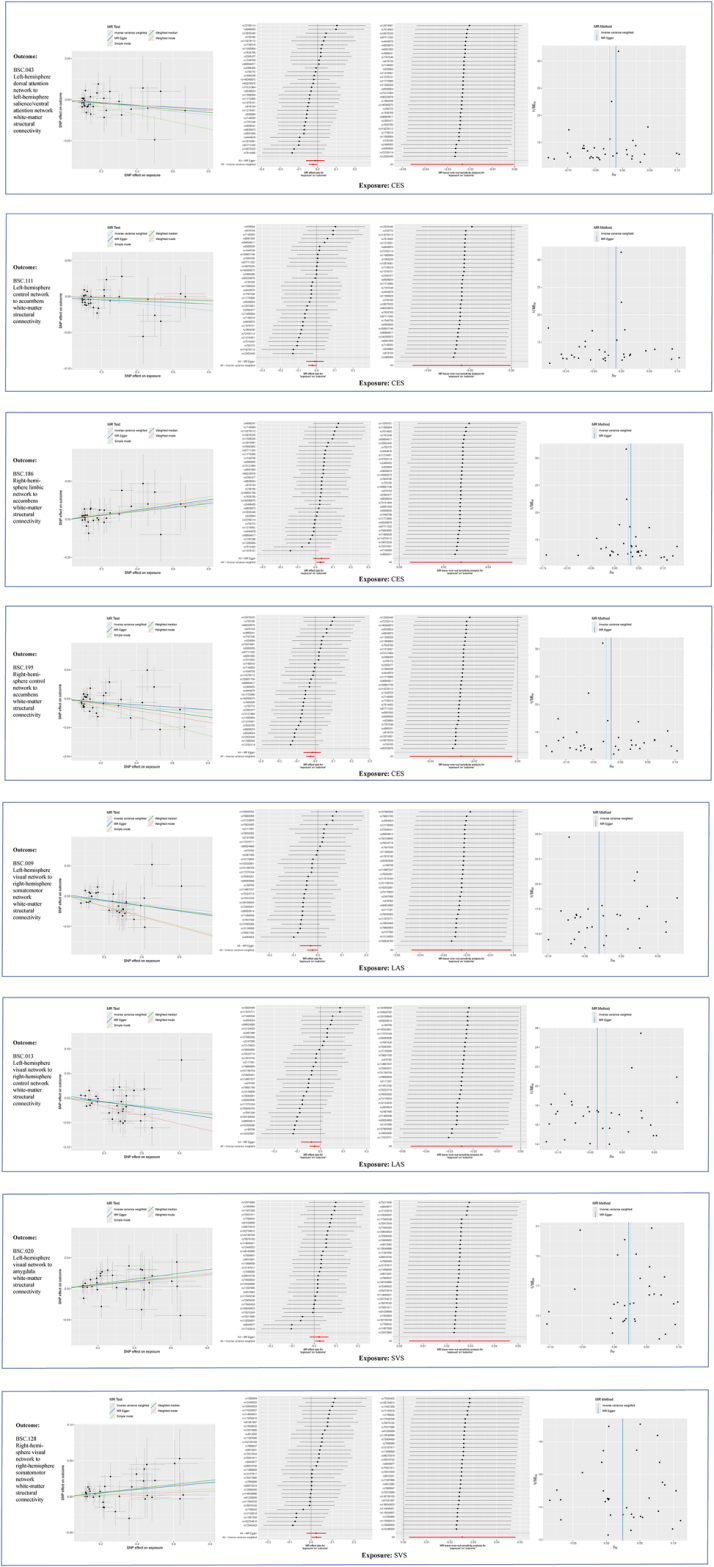
Scatterplots, forest plots, LOO analysis, and funnel plots of reverse MR analyses between stroke and BSCs.

Genome-wide aggregated data for BSC were obtained by accessing the GWAS directory of the European Bioinformatics Institute (https://www.ebi.ac.uk/gwas). Structural connectivity (SC) traits were sourced from Wainberg et al. (2024). Whole-brain diffusion MRI tractograms for 26,300 UK Biobank participants were reconstructed with the MRtrix3 standard pipeline followed by SIFT2 reweighting. The study defined three hemispheric measures of connectivity [intra-left hemisphere (LH), intra-right hemisphere (RH), and interhemisphere; Wainberg et al., 2024 ]. Thirty-one macroscale regions (seven canonical cortical networks plus 14 subcortical structures) were parcellated, yielding 206 log10-transformed, SIFT2-weighted streamline count edges that capture intra-LH, intra-RH, and interhemispheric WM connectivity (Fig. 2). Full preprocessing and quality-control procedures (motion thresholds and dMRI QC scoring) are detailed in Wainberg et al. (2024).

The GWAS data of intracerebral hemorrhage (ICH) was provided by FinnGen (*n*Case = 4,056; *n*Controls = 371,717; Kurki et al., 2023). The FinnGen study is a large-scale genomics initiative that has analyzed >500,000 Finnish biobank samples and correlated genetic variation with health data to understand disease mechanisms and predispositions. The project is a collaboration between research organizations and biobanks within Finland and international industry partners. Ischemic stroke (IS) abstract-level data on the IS subtypes were obtained from the MEGASTROKE Consortium genome-wide association meta-analysis (Malik et al., 2018). IS (*n*Case = 342,17; *n*Controls = 406,111) and the IS subcategories are as follows: large-artery atherosclerotic stroke (LAS; *n*Case = 4,373; *n*Controls = 146,392); small-vessel IS (SVS; *n*Case = 5,386; *n*Controls = 192,662); and cardioembolic IS (CES; *n*Case = 7,193; *n*Controls = 204,570).

### IV selection

The IVs associated with BSC were evaluated from multiple perspectives through rigorous screening criteria. The significance threshold, *P*, was reduced to <5 × 10^−6^ given the moderate number of BSC-related SNPs. Then, the linkage disequilibrium (LD; *r*^2^ < 0.001; kb = 10,000) was reduced. *R*^2^ and the *F* statistic were calculated for each SNP to eliminate the bias caused by poor instruments. The methods for calculating *R*^2^ and the *F* statistic (Burgess and Thompson, 2011) are as follows:

$$F=\frac{N-k-1}{k}\;*\;\frac{R^{2}}{1-R^{2}},$$

$$R^{2}=\frac{2\;*\;\beta^{2}\;*\;\mathrm{EAF}\;*\;(1-\mathrm{EAF})}{\left[2\;*\;\beta^{2}\;*\;\mathrm{EAF}\;*\;(1-\mathrm{EAF})+2\;*\;{\left(\mathrm{se}(\beta )\right)}^{2}\;*\;N\;*\;\mathrm{EAF}\;*\;(1-\mathrm{EAF})\right]}.$$

SNPs with an *F* < 10 were defined as poor genetic variants and removed (Burgess et al., 2013). Next, BSC-related SNPs were extracted from the results to coordinate the SNPs between exposure and outcome, and the SNPs with incongruent palindromic effects and alleles were removed. Then, to satisfy hypothesis (3), all instrumental SNPs were screened for associations with stroke-related outcomes. No SNPs met the exclusion threshold (*p* < 5 × 10^−5^), so no variants were removed. Finally, MR analyses were performed for exposures with >2 SNPs (Yun et al., 2023).

### Statistical and sensitivity analyses

The relationships of BSC and stroke were evaluated based on inverse-variance weighted (IVW) results (Pierce and Burgess, 2013; Burgess et al., 2015). MR-Egger, weighted median, weighted mode, and simple modes were used as complementary analyses to obtain more reliable results. The Bonferroni’s correction significance threshold was applied for MR analysis to determine the relationship between BSC and stroke (Curtin and Schulz, 1998) and calculated as 4.85 × 10^−5^ (*p* < 0.05, accounting for 206 exposures and five outcomes). The suggested association results were significant before multiple-comparison adjustment (*p* < 0.05) but not significant after multiple-comparison adjustment (*p* < 4.85 × 10^−5^, Bonferroni’s adjustment). After adjustment, a *p* < 4.85 × 10^−5^ was considered statistically significant.

In this study, four sensitivity analysis methods were used to detect and correct for heterogeneity and pleiotropy, including the Cochran *Q* test, MR-Egger intercept test, MR-PRESSO, and radial MR. A *p* < 0.05 on the Cochran *Q* test was considered heterogeneity of the results (Greco et al., 2015). The MR-Egger intercept was calculated to assess directional pleiotropy and bias due to an invalid IV (Burgess and Thompson, 2017). Radial MR was subsequently performed for outlier identification (Bowden et al., 2018). MR analysis was repeated after heterogeneous SNPs were eliminated. Finally, MR-PRESSO was used to recheck for the presence of heterogeneous SNPs (Verbanck et al., 2018). A leave-one-out (LOO) analysis was performed to assess whether the results were heavily influenced by a single SNP by discarding each SNP in turn, followed by an MR analysis (Burgess and Thompson, 2017).

Although multiple sensitivity analyses were refined to eliminate SNPs that did not conform to the hypothesis, there may still be a small number of residual confounding SNPs. The LDlink website (https://ldlink.nih.gov/?tab=home) was searched for IVs in BSCs (threshold, *R*^2^ = 0.1 ± 500,000; Machiela and Chanock, 2015) to determine whether each SNP is associated with known risk factors for stroke, such as cigarette smoking, high blood pressure, and diabetes (GBD 2019 Stroke Collaborators, 2021). SNPs associated with the abovementioned confounders (*p* < 1 × 10^−5^) were removed, and MR analysis was repeated to verify the reliability of the results.

In summary, BSCs with a potential relationship to stroke across multiple criteria were rigorously screened, as follows: (1) The *p* value of the preliminary analysis was significant (*p* < 0.05 derived from IVW). (2) The direction and amplitude of the five MR methods were consistent. (3) There was no heterogeneity or horizontal pleiotropy in the MR results. (4) The MR estimates were not severely disturbed by a single SNP. (5) Confounding factors were excluded.

### Evaluation of genetic correlation and directivity

Although we excluded SNPs associated with stroke when selecting IVs, SNPs that are not associated may also mediate the genetics of stroke. Therefore, LD score (LDSC) regression was applied to examine the genetic correlation between screened BSCs and AS (Bulik-Sullivan et al., 2015).

To further verify the correctness of the assumed direction under the relaxed IV selection threshold (*p* < 5 × 10^−6^), the Steiger directionality test was applied. The Steiger test indicated that the variance explained in the exposure exceeded that in the outcome for all BSC–stroke associations that passed multiple testing correction, supporting the assumed direction from BSC traits to stroke risk. This consistency provides additional assurance that the relaxed threshold did not compromise instrument validity (Hemani et al., 2017).

### Colocalization

Genes that have important regulatory roles in BSC were identified from previous studies, and colocalization analysis was performed using the coloc R package (Giambartolomei et al., 2014) to further investigate whether the association between BSC and stroke is driven by loci within genomic regions. Coloc assumes that there is at most one association per trait in the test area, and approximate Bayes factor computation generates all possible posterior probabilities between the two traits (PP), as follows: (1) H0, SNPs in the region were not associated with any phenotype; (2) H1, SNPs in the region were associated with phenotype 1 but not phenotype 2; (3) H2, SNPs in the region were associated with phenotype 2 but not phenotype 1; (4) H3, Phenotypes 1 and 2 are significantly associated with SNP sites in a genomic region, driven by different variation sites; and (5) H4, Phenotypes 1 and 2 are significantly associated with SNP sites in a genomic region and are driven by the same mutation site. Each type of PP is represented by PP_0_, PP_1_, PP_2_, PP_3_, and PP_4_. PP_4_ > 90% is considered a strong support for copositioning (Giambartolomei et al., 2014). Genomic regions were defined within 100 kb of the instrumental SNP variables. The colocalization analysis was performed using the default priors (*P*1 = 1 × 10^−4^, *P*2 = 1 × 10^−4^, and *P*12 = 1 × 10^−5^).

### Multivariable MR

Multivariable MR (MVMR) was introduced to assess the direct impact of each exposure (independent of any other exposures) on the results, which can correct for the interaction of genetic variation between exposures by merging multiple exposures that may interact (Burgess and Thompson, 2015). MVMR was performed using IVW and MR-PRESSO. Then, regression of all exposed SNPs was performed by weighting the inverse variance of the results. MR-PRESSO can remove outliers to correct for IV pleiotropy. Bonferroni’s correction was also performed for the MVMR analysis results.

### Reverse MR analysis

Reverse MR analysis was performed with various strokes as exposure and BSC as outcome to explore the relationship between stroke and BSC. The *p* was adjusted to <5 × 10^−6^, and the LD was removed (*r*^2 ^< 0.001; kb = 10,000) to extract stroke-related IVs.

## Results

### Preliminary analysis

A total of 206 BSCs were used for MR analysis, and the quality of the filtered IV was strictly controlled with 5–43 SNPs (the genetic agent for BSC.062 consisted of 5 SNPs; BSC.029 produced the most genetic agents, 43 SNPs). The *F* statistic for all BSC-related SNPs was >10.

**Figure 3.**
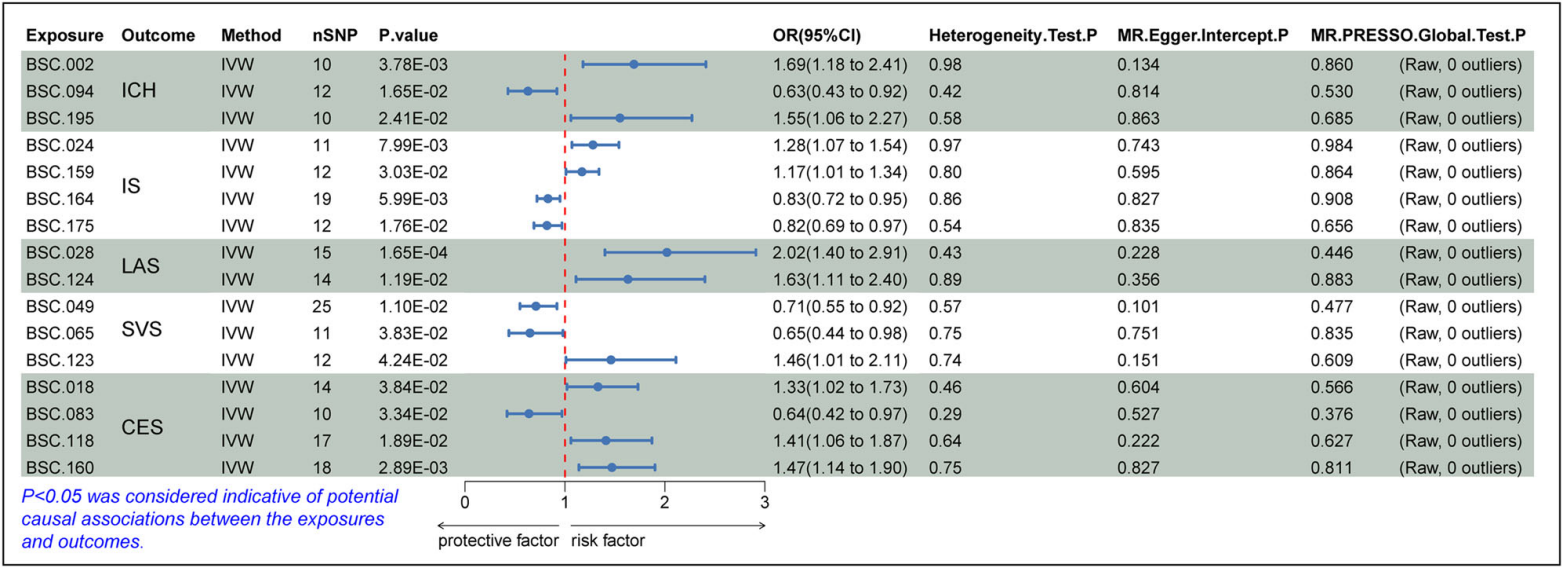
Forest plot for the relationship between BSCs and stroke derived from IVW analysis. CI, confidence interval; OR, odds ratio; *p*_bfr, *p* value after Bonferroni’s correction.

Two-sample MR analysis was performed using 206 BSCs as exposures with ICH, IS, LAS, SVS, and CES, and 6 BSCs were preliminarily identified to have a close relationship with ICH. Nine types of BSCs had a potential relationship with IS. Eight types of exposures had a potential relationship with LAS. There were also eight closely related exposures for SVS. Seven types of BSCs were associated with CES. Then sensitivity and confounding analyses were completed and SNPs with heterogeneity, horizontal pleiotropy, and confounding effects were excluded. The analysis was repeated. A total of three BSCs were exposed with positive results for ICH: LH visual network-to-LH somatomotor network WM-SC (BSC.002, OR = 1.69; 95% CI, 1.18–2.41, *p* = 3.78 × 10^−3^); LH limbic network-to-amygdala WM-SC (BSC.094, OR = 0.63; 95% CI, 0.43–0.92; *p* = 1.65 × 10^−2^), and RH control network-to-accumbens WM-SC (BSC.195, OR = 1.56; 95% CI, 1.06–2.27; *p* = 2.41 × 10^−2^). There are four types of BSCs exposed with positive results for IS: LH somatomotor network-to-LH salience/ventral attention network WM-SC (BSC.024, OR = 1.28; 95% CI, 1.07–1.54; *p* = 7.99 × 10^−3^); RH dorsal attention network (DAN)-to-thalamus WM-SC (BSC.159, OR = 1.17; 95% CI, 1.02–1.34; *p* = 3.03 × 10^−2^); RH DAN-to-amygdala WM-SC (BSC.164, OR = 0.83; 95% CI, 0.72–0.95; *p* = 5.99 × 10^−3^); and RH salience/ventral attention network-to-amygdala WM-SC (BSC.175, OR = 0.82; 95% CI, 0.69–0.97; *p* = 1.76 × 10^−2^). LH somatomotor network-to-RH visual network WM-SC (BSC.028, OR = 2.02; 95% CI, 1.40–2.91; *p* = 1.65 × 10^−4^) and LH default mode network-to-hippocampus WM-SC (BSC.124, OR = 1.63; 95% CI, 1.12–2.40; *p* = 1.19 × 10^−2^) correspond to LAS. Three BSCs, including LH DAN-to-RH DAN WM-SC (BSC.049, OR = 0.71; 95% CI, 0.55–0.93; *p* = 1.10 × 10^−2^), LH salience/ventral attention network-to-RH visual network WM-SC (BSC.065, OR = 0.65; 95% CI, 0.44–0.98; *p* = 3.83 × 10^−2^), and LH default mode network-to-pallidum WM-SC (BSC.123, OR = 1.46; 95% CI, 1.01–2.11; *p* = 4.24 × 10^−2^), have a potential relationship with SVS. CES-related BSCs include the LH visual network-to-pallidum WM-SC (BSC.018, OR = 1.33; 95% CI, 1.02–1.73; *p* = 3.84 × 10^−2^), LH limbic network-to-RH somatomotor network WM-SC (BSC.083, OR = 0.64; 95% CI, 0.42–0.97; *p* = 3.34 × 10^−2^), LH default mode network-to-RH control network WM-SC (BSC.118, OR = 1.41; 95% CI, 1.06–1.87; *p* = 1.89 × 10^−2^), and RH DAN-to-caudate WM-SC (BSC.160, OR = 1.47; 95% CI, 1.14–1.90; *p* = 2.89 × 10^−3^). In summary, the IVW estimates were significant (*p* < 0.05), and the direction and amplitude of IVW, MR-Egger, WM, and WMSM estimates were consistent (Fig. 3).

**Figure 4.**
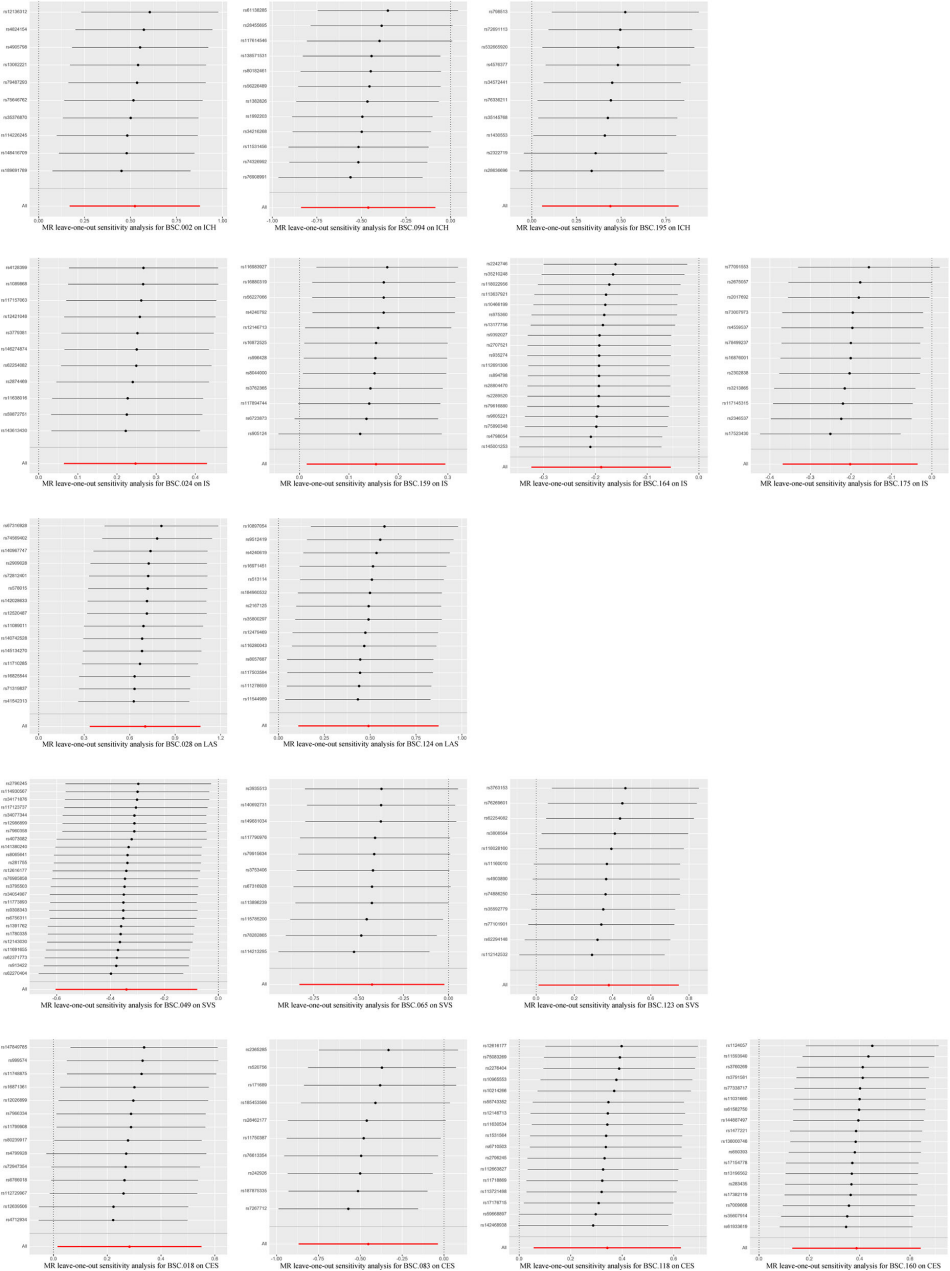
LOO analysis of MR analyses between BSCs and stroke.

**Figure 5.**
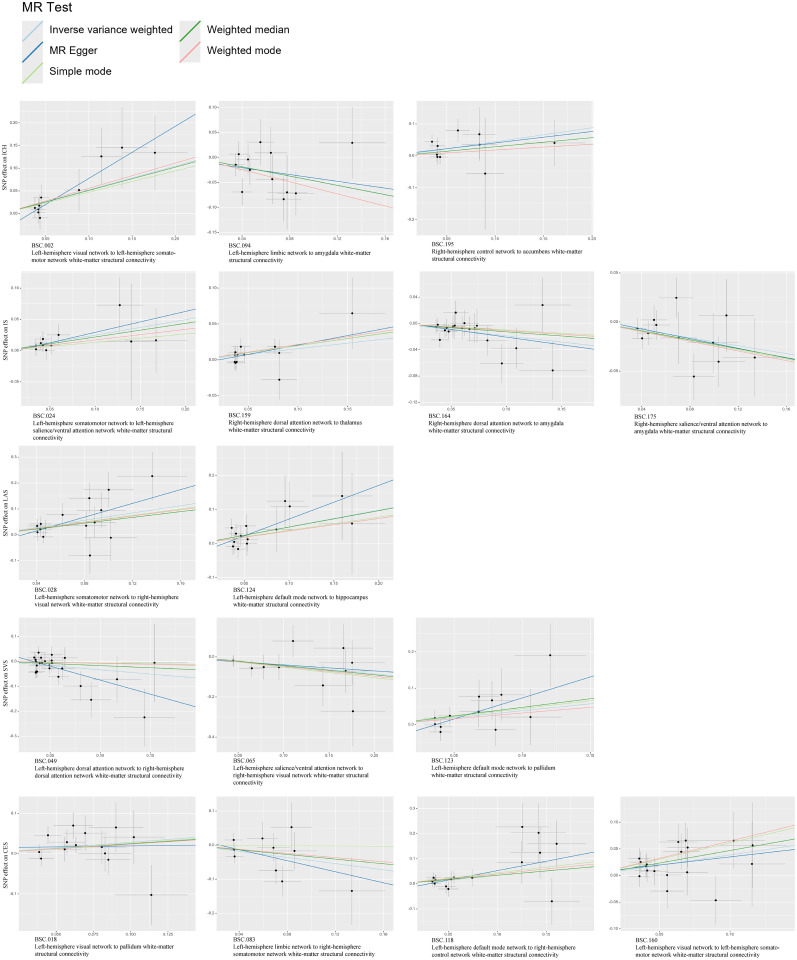
Scatterplots of MR analyses between BSCs and stroke.

**Figure 6.**
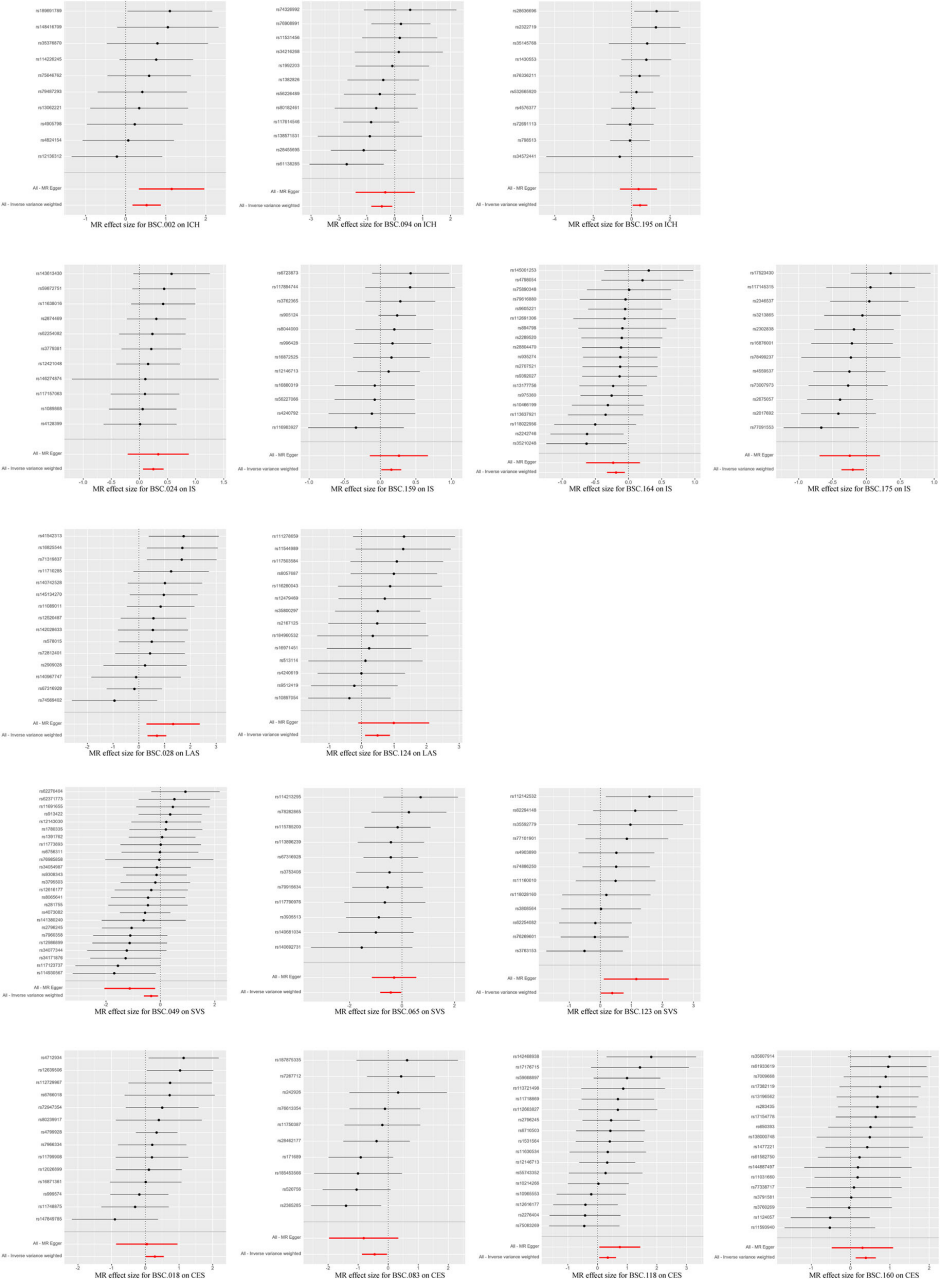
Forest plots of MR analyses between BSCs and stroke.

**Figure 7.**
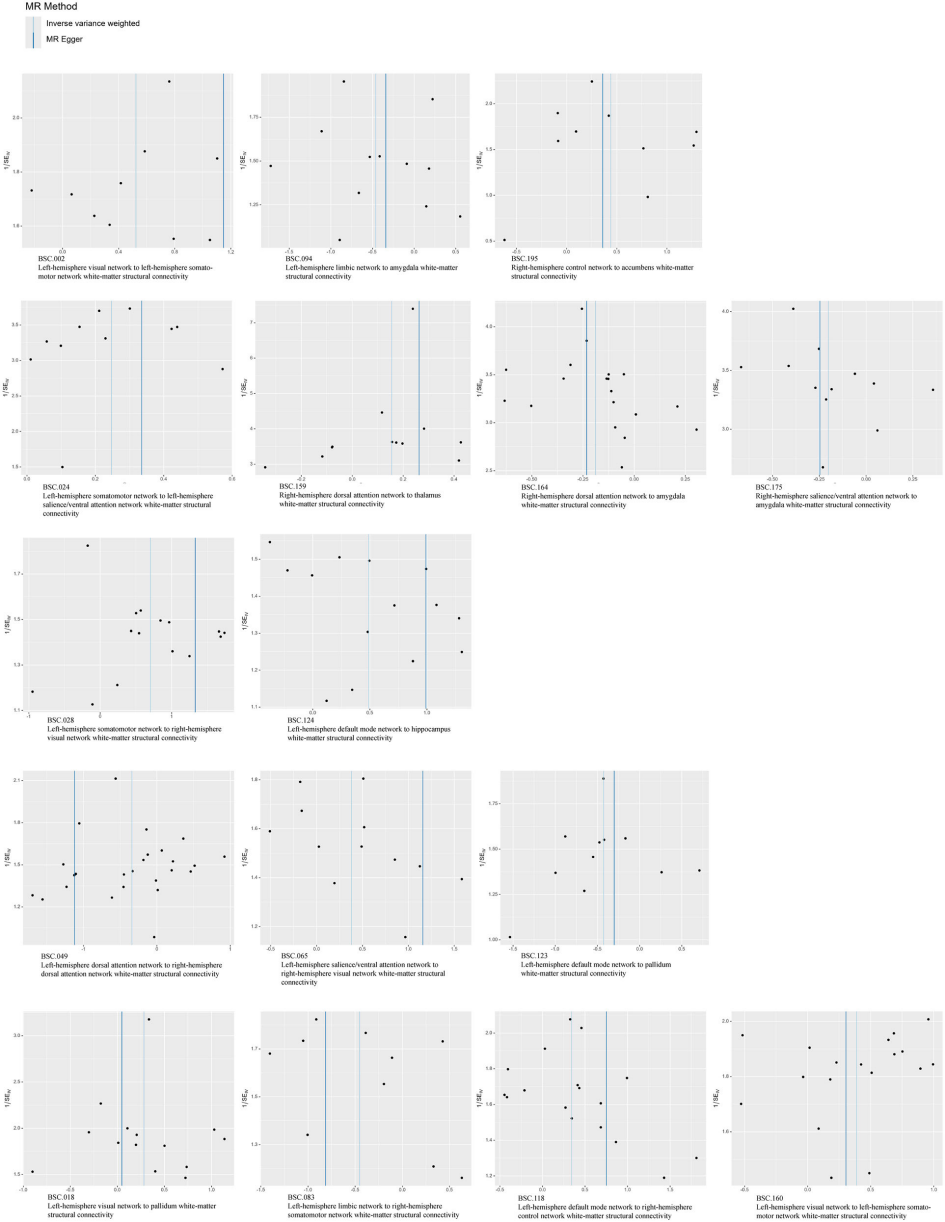
Funnel plots of MR analyses between BSCs and stroke.

The existence of heterogeneous SNPs was not observed after radial MR identification and removal of outliers. Strong evidence was shown for the absence of heterogeneity and pleiotropy with the Cochran *Q* (*p* > 0.05) and MR-Egger intercept tests (*p* > 0.05). The LOO analysis results supported the finding that a single SNP does not lead to bias in MR estimates (Fig. 4). Figures 5–7 are the scatter, forest, and funnel plots of the associations between BSCs and stroke, respectively. The sensitivity analysis ruled out SNPs that violated the estimated values. To satisfy hypothesis 2 (IVs are not associated with confounders), we examined one-by-one whether all SNPs associated with BSCs were associated with common risk factors for stroke (smoking, hypertension, and diabetes). After removing these SNPs, the estimated values remained significant. Overall, none of these associations survived Bonferroni’s correction [*p* value after Bonferroni’s correction (*p.bfr*) > 0.05; S4]. Nevertheless, traits with an uncorrected *p* < 0.05 were carried forward as exploratory candidate exposures for MVMR.

### Evaluation of genetic correlation and directivity

Ldsc-based estimates indicated that there was little genetic correlation between BSC and stroke type, as follows: BSC.002-ICH (*R_g_* = −0.127; Se = 0.132; *p* = 0.335); BSC.094-ICH (*R_g_* = 0.200; Se = 0.210; *p* = 0.341); BSC.195-ICH (*R_g_* = 0.188; Se = 0.159; *p* = 0.236); BSC.024-IS (*R_g_* = 0.009; Se = 0.101; *p* = 0.932); BSC.159-IS (*R_g_* = 0.081; Se = 0.092; *p* = 0.377); BSC.164-IS (*R_g_* = −0.028; Se = 0.092; *p* = 0.763); BSC.175-IS (*R_g_* = 0.024; Se = 0.132; *p* = 0.857); BSC.028-LAS (*R_g_* = −0.045; Se = 0.272; *p* = 0.869); BSC.124-LAS (*R_g_* = −0.301; Se = 0.184; *p* = 0.102); BSC.049-SVS (*R_g_* = −0.265; Se = 0.144; *p* = 0.065); BSC.065-SVS (*R_g_* = 0.167; Se = 0.176; *p* = 0.341); BSC.123-SVS (*R_g_* = −0.131; Se = 0.153; *p* = 0.390); BSC.018-CES (*R_g_* = −0.113; Se = 0.128; *p* = 0.379); BSC.083-CES (*R_g_* = 0.117; Se = 0.145; *p* = 0.420); BSC.118-CES (*R_g_* = −0.062; Se = 0.102; *p* = 0.543); and BSC.160-CES (*R_g_* = −0.040; Se = 0.131; *p* = 0.761). The SNP heritability of the 16 BSCs included in subsequent analyses based on LDSC were then estimated with heritability (the proportion of variance attributable to the genome-wide SNPs) ranging from 0.0559 (BSC.175) to 0.1915 (BSC.118). In addition, Steiger's test showed that the close relationship between BSC and corresponding types of strokes was not undermined by a reverse way.

### Colocalization analysis

**Figure 8.**
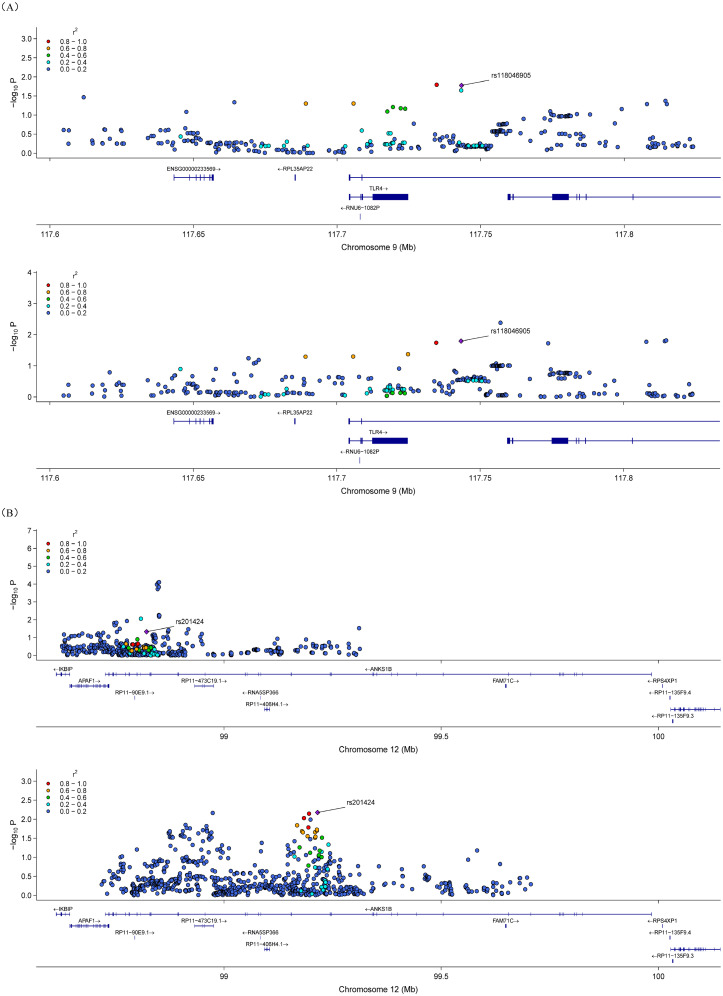
Colocalization analysis depicting the genomic regions and risky SNPs associated with both the BSCs and stroke. The *x*-axis shows the position within the genome (building Hg19) and *y*-axis denotes the −log_10_(*P*) for the association. Color denotes the LD between different variants. *A*, The colocalization analysis result for the LH limbic network-to-amygdala WM-SC (BSC.094) and ICH indicated the SNP, rs118046905 (*p* = 1.61 × 10^−2^ GWAS for BSC.094 and *p* = 1.68 × 10^−2^ GWAS for ICH), as a SNP at high risk. *B*, The colocalization analysis result for RH DAN-to-caudate WM-SC (BSC.160) and CES indicated the SNP, rs201424 (*p* = 4.71 × 10^−2^ GWAS for BSC.160 and *p* = 6.67 × 10^−3^ GWAS for CES), as a SNP at high risk.

Shared association between BSC and stroke was tested using coloc, and these SNPs had a putative relationship in the MR analysis. The analysis showed that BSC.094 was colocated with ICH (PP_4_ = 0.999) in a region of chromosome 9 (Fig. 8*A*) and BSC.160 and CES (PP_4_ = 0.994) in a region of chromosome 12 (Fig. 8*B*). The candidate risk SNPs for these two colocalized regions were rs118046905 and rs201424, respectively (Fig. 8).

### MVMR

**Figure 9.**
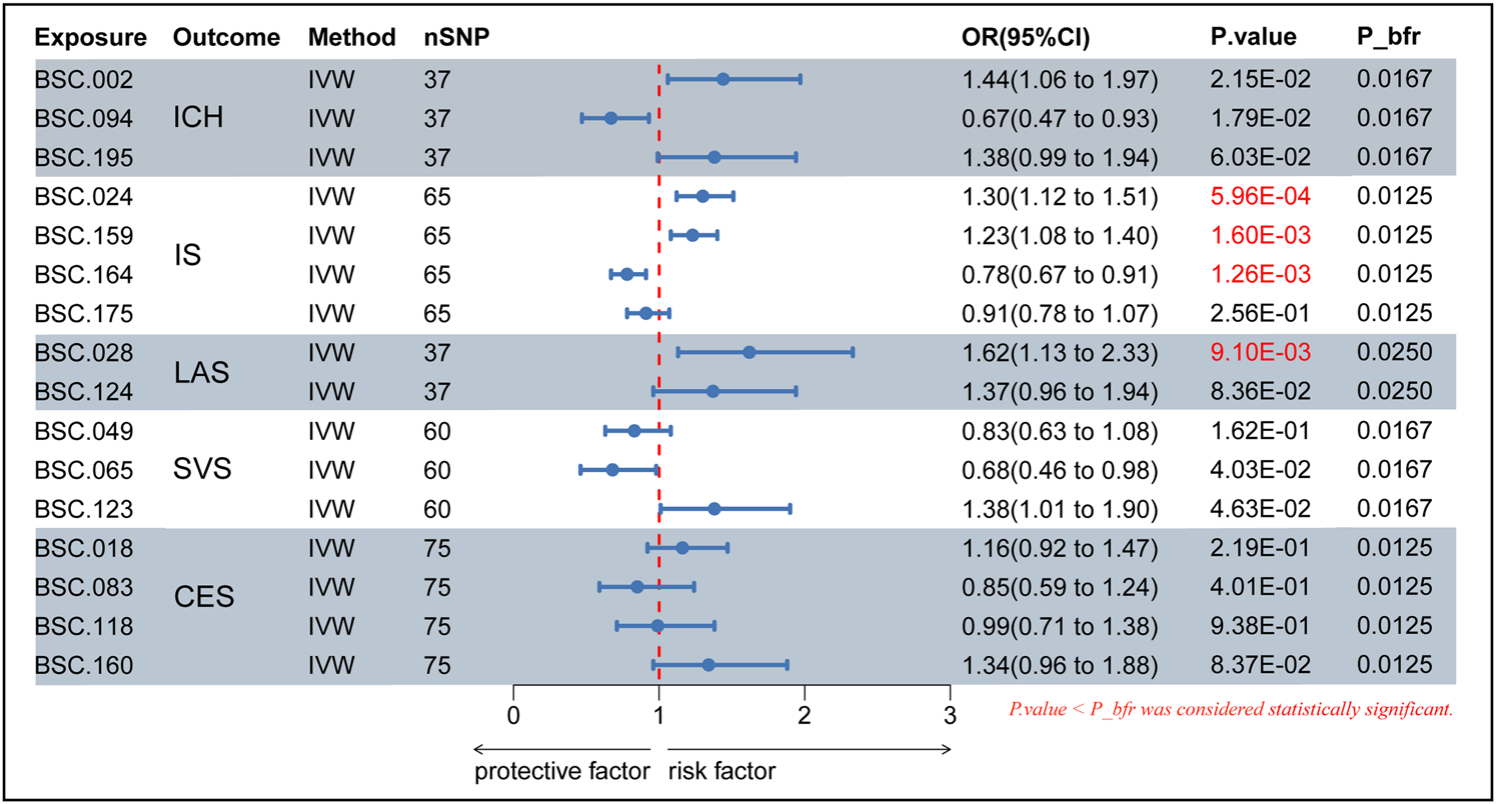
MVMR analysis of the final BSCs. 95% CI, 95% confidence interval; IVW, inverse-variance weighted; *p*_bfr, the *p* value after Bonferroni’s correction.

MVMR analyses were performed and the results were Bonferroni-corrected to adjust for interactions between exposures. At a *p* < *p.bfr* (0.05/number of exposures/number of outcomes, significance threshold of Bonferroni’s correction), the data were statistically significant. BSC.024 (OR = 1.30; 95% CI, 1.12–1.51; *p* = 5.96 × 10^−4^; *p.bfr* = 0.0125), BSC.159 (OR = 1.23; 95% CI, 1.08–1.40; *p* = 1.60 × 10^−3^), and BSC.164 (OR = 0.78; 95% CI, 0.67–0.91; *p* = 1.26 × 10^−3^) directly affected IS independent of other exposures based on the IVW estimation in MVMR (Fig. 9). BSC.028 (OR = 1.62; 95% CI, 1.13–2.33; *p* = 9.10 × 10^−3^; *p.bfr* = 0.025) was an independent factor influencing LAS. IVW and MR-Egger tests showed no heterogeneity or horizontal pleiotropy.

### Reverse MR result

Reverse MR did not identify any associations that survived Bonferroni’s correction. However, eight BSC–stroke subtype pairs showed nominal significance (uncorrected *p* < 0.05; Fig. 2). There was a potential for reverse relationship between the LH DAN-to-LH salience/ventral attention network WM-SC (OR = 0.98; 95% CI, 0.95–1.00; *p* = 0.046), LH control network-to-accumbens WM-SC (OR = 0.98; 95% CI, 0.95–1.00; *p* = 0.046), RH limbic network-to-accumbens WM-SC (OR = 1.03; 95% CI, 1.00–1.05; *p* = 0.018), RH control network-to-accumbens WM-SC (OR = 0.97; 95% CI, 0.95–1.00; *p* = 0.030), and CES. There was a reverse correlation between the LAS and LH visual network-to-RH somatomotor network WM-SC (OR = 0.98; 95% CI, 0.96–1.00; *p* = 0.042) and LH visual network-to-RH control network WM-SC (OR = 0.98; 95% CI, 0.95–1.00; *p* = 0.023). The LH visual network-to-amygdala WM-SC (OR = 1.03; 95% CI, 1.00–1.05; *p* = 0.019) and RH visual network-to-RH somatomotor network WM-SC (OR = 1.03; 95% CI, 1.00–1.05; *p* = 0.030) was inversely correlated with SVS. This revision offers a balanced presentation, acknowledging statistical limitations while recognizing potential biological relevance.

## Discussion

The current study showed that the LH visual network-to-LH somatomotor network WM-SC and RH control network-to-accumbens WM-SC increased the potential risk of ICH occurrence. The LH limbic network-to-amygdala WM-SC was related to a low risk of ICH, while the LH DAN-to-RH DAN WM-SC and LH salience/ventral attention network-to-RH visual network WM-SC had a close association with a potentially low SVS risk. The LH default mode network-to-pallidum WM-SC was related to a high risk of ICH. The LH visual network-to-pallidum WM-SC, LH default mode network-to-RH control network WM-SC, and RH DAN-to-caudate WM-SC suggested a potentially high-risk correlation with CES. The LH limbic network-to-RH somatomotor network WM-SC indicated a lower risk of CES. However, multivariate analysis of the above items did not show a significant independent association.

The LH somatomotor network-to-LH salience/ventral attention network WM-SC and RH DAN-to-thalamus WM-SC showed a higher potential risk of IS occurrence, while the RH DAN-to-amygdala WM-SC and RH salience/ventral attention network-to-amygdala WM-SC had a lower potential risk of IS occurrence. After perfecting multivariate analysis the following three BSCs still directly affected IS independent of other exposures: LH somatomotor network-to-LH salience/ventral attention network WM-SC; RH DAN-to-thalamus WM-SC; and RH DAN-to-amygdala WM-SC. The LH somatomotor network-to-RH visual network WM-SC and LH default mode network-to-hippocampus WM-SC had a potential positive correlation with LAS. Subsequent multivariate analysis supported the finding that the LH somatomotor network-to-RH visual network WM-SC had a significant independent effect on LAS.

Colocalization analyses were performed to determine whether BSC-stroke associations arose from shared variants at risk. The LH limbic network-to-amygdala BSC shared a common locus with ICH, and the RH dorsal attention-to-caudate BSC shared a locus with CES. These results implied that in some cases, genetic variants may jointly influence both BSC and stroke through overlapping biological pathways.

The neurovascular coupling hypothesis posits a dynamic relationship between local neural activity and cerebral blood flow (Iadecola, 2017). Under this framework, greater connectivity implies more frequent or sustained neural signaling, which places higher metabolic demands on the regional vasculature (Wang et al., 2023). Highly connected regions may be at greater risk of ischemia if this coupling becomes inefficient due to chronic vascular comorbidities. Hypertension, hyperglycemia, and cigarette smoking can exacerbate local cerebral vascular stenosis and thrombosis (Maraka et al., 2014; O'Donnell et al., 2016; Xiong et al., 2016; Hannawi et al., 2018). Studies have shown that hypertension is genetically associated with multiple WM tracts, including the superior longitudinal fasciculus, internal capsule, and external capsule (Hannawi et al., 2018), while cigarette smoking and type 2 diabetes influence corticospinal tracts (Maraka et al., 2014; Xiong et al., 2016; Wassenaar et al., 2019). When combined with the neurovascular coupling hypothesis, we speculated that in regions of high connectivity and demand, increased perfusion may deprive other regions, leading to ischemia and stroke. The current study supported this hypothesis; specifically, three BSCs demonstrated independent risk for the IS or LAS.

**Figure 10.**
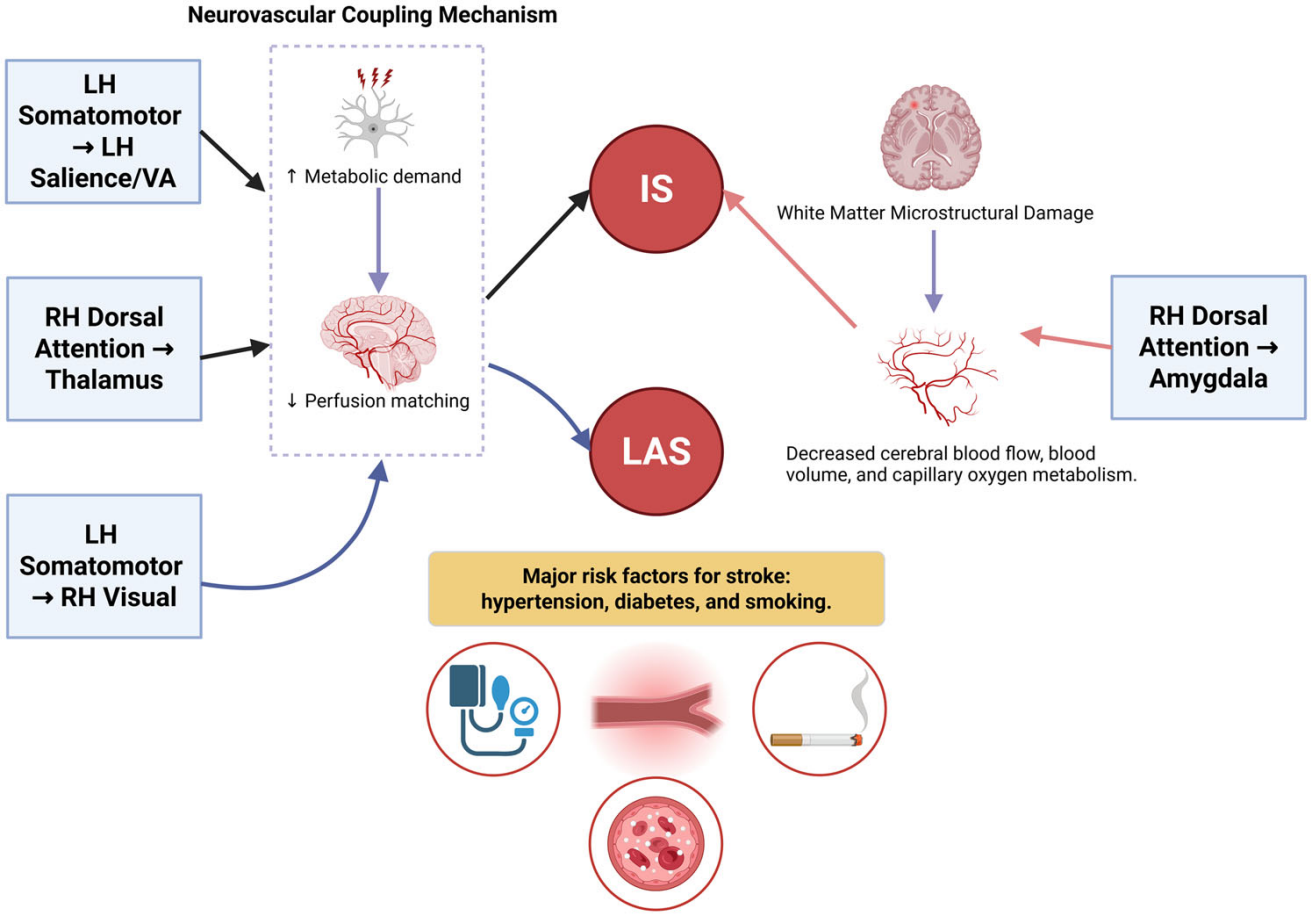
Mechanisms linking BSC to stroke risk. This schematic diagram illustrates how WM structural connectivity (BSC) influences stroke risk, focusing on IS and large-artery stroke (LAS). The diagram shows positive and negative associations between BSC patterns and stroke risk. Increased metabolic demand in brain regions with strong connectivity (e.g., LH somatomotor-to-LH salience/VA and RH dorsal attention-to-thalamus) leads to perfusion mismatch (IS risk). RH dorsal attention-to-amygdala is inversely associated with IS, suggesting a protective effect. LH somatomotor-to-RH visual network is positively correlated with LAS. The figure also highlights the effects of WM damage, decreased blood flow, and capillary oxygen metabolism on stroke risk, with major risk factors, like hypertension, diabetes, and cigarette smoking, contributing to vascular dysfunction and elevated stroke risk.

Changes in WM microstructure are believed to precede irreversible lesions and are associated with decreased cerebral blood flow, blood volume, and oxygen metabolism (Kaffashian et al., 2016; Yu et al., 2023). The finding that decreased LH default mode network-to-hippocampus WM-SC connectivity was associated with higher IS risk may reflect such a mechanism. This highlights the clinical relevance of BSC screening in identifying at-risk individuals. To visualize the mechanisms linking BSC to stroke risk, we have provided a schematic diagram that highlights the key associations with IS and LAS (Fig. 10).

In this study we found significant associations between BSC and stroke subtypes, including IS and LAS. Specifically, the enhanced connectivity between the LH somatomotor network and LH salience/ventral attention network was associated with increased IS risk. These two networks are involved in emotion regulation, cognitive control, and attentional processing. The LH somatomotor network, which includes the somatosensory and motor areas, is crucial for motor control, while the LH salience/ventral attention network, consisting of the anterior insula and dorsal anterior cingulate cortex, has a role in processing emotions and regulating attention (Martino et al., 2016; Akiki et al., 2017, 2025). The observed increase in connectivity between these networks may indicate heightened metabolic demand, particularly in the context of vascular risk factors, such as hypertension and diabetes. Under these conditions, the enhanced neural activity may lead to insufficient blood supply, which increases the risk of ischemic damage and IS. In addition, the involvement of the insula and prefrontal cortex in emotional and self-regulation suggests that these regions may impact neurovascular coupling mechanisms. Over time, this compensatory mechanism may increase blood flow imbalance, further elevating the risk of stroke.

The DAN, which includes the intraparietal sulcus and the frontal eye field, is primarily involved in goal-directed attention and visuospatial processing (Kincade et al., 2005; Farrant and Uddin, 2015). The thalamus has an essential role in integrating sensory information. Enhanced SC of the DAN-to-the thalamus likely reflects an increased demand for visuospatial attention, which can lead to increased metabolic activity in these regions. In the presence of chronic vascular risk factors, such as hypertension and diabetes, the enhanced connectivity may increase the risk of ischemic damage due to insufficient blood flow. In the current study, the enhanced connectivity between the RH DAN and amygdala had a negative association with IS. The DAN is primarily involved in visuospatial attention and goal-directed regulation, while the amygdala has a crucial role in emotional processing and autonomic regulation (Tang et al., 2020). The observed enhancement in the connectivity between these regions could indicate a protective role in regulating emotional stress responses and attention distribution, especially under conditions of vascular risk factors, such as hypertension and diabetes. These vascular risk factors may not only be related to the structural damage to WM but may also impair emotional regulation, leading to maladaptive stress responses and increased susceptibility to ischemic damage. The enhanced connectivity between RH DAN and amygdala could be a compensatory mechanism in response to these chronic stressors, helping to mitigate ischemic damage.

The LH somatomotor network-to-RH visual network WM-SC had a positive association with LAS, indicating that the interaction between motor regulation and visuospatial processing may increase stroke risk. The LH somatomotor network is involved in motor control, while the RH visual network processes visual spatial information (Yang et al., 2015). The enhanced connectivity between these two networks may represent a synergistic interaction between visual and motor regulation, increasing the metabolic demand in brain regions, which in turn raises the risk of LAS.

Genetic colocalization studies suggest that stroke-associated SNPs often affect WM regions, such as the corpus callosum, internal capsule, and superior longitudinal fasciculus, which are also implicated in metabolic syndromes, like diabetes and hypertension (Zhao et al., 2021). These three colocalized regions are also associated with type 2 diabetes, obesity, and hypertension, suggesting that the genetic relationship between WM microstructure and stroke may be mediated in part by vascular risk factors. Moreover, stroke was genetically related to the corpus callosum, radiocarina, inner capsule, outer capsule, upper longitudinal tract, supraconcipital tract, and uncinate tract sharing genetic loci (Zhao et al., 2021). The colocalization analysis confirmed two SNPs (rs118046905 and rs201424) potentially mediating BSC.094-ICH and BSC.160-CES associations, respectively. These loci remain functionally unexplored and warrant further biological validation.

A potential reverse relationship was demonstrated between eight BSCs and IS subtypes based on the reverse MR analysis, although these results were not significant after Bonferroni’s adjustment. The effects of a stroke on BSC are unavoidable and usually occur within minutes of onset. At the same time, BSC expressed as small worldness and modularity, which makes BSC into two forms (proximal and distal connectivity) at the same time (Park and Friston, 2013; Silasi and Murphy, 2014). The ischemic area is regarded as the necrotic core after IS, and various effects that lead to increased expression of neuroprotective proteins, nerve growth factors, and neurotransmitters not only occur near the lesion but also in the distant region and the contralateral hemisphere, which also indicates that stroke can have damaging effects on the SC between different regions (Sotelo et al., 2020; Yourganov et al., 2021; Zhao et al., 2023; Cao et al., 2025). Furthermore, it has been suggested that patients with significant degeneration of motor fibers in the corpus callosum are also more likely to exhibit pathologically enhanced motor activity in the contralateral hemisphere (Grefkes and Fink, 2014), so the overactivity in the unaffected hemisphere may be affected by interhemispherical inhibition disorder. The increased SC related to elevated stroke risk may be a compensatory effect for maintaining neural functional activity in the early stages of stroke.

This MR study had several advantages. First, the relationship between 206 BSCs and various types of stroke, which has not been fully studied by previous studies, was examined for the first time. Second, the current study effectively avoided the drawbacks of confounding factor uncertainty and reverse relationship in traditional observational research methods. Third, the abstraction-level data came from the most recent GWAS for populations of European ancestry, which improved the statistical power of the study and provided compelling evidence of an association. Fourth, several alternative methods were used to estimate the possible pleiotropic bias and obtained consistent effect estimates, which confirms the robustness of the findings. Finally, the heritability of IVs and the genetic association between BSC and stroke using LDSC were evaluated, which made the MR estimate more convincing. Colocalization analyses were also performed to provide evidence at the genetic level.

We now emphasize that specific BSC profiles, particularly those showing independent associations with IS and LAS, may serve as early indicators of stroke susceptibility. Therefore, the attention can be paid to the BSC profiles, especially those independently associations with IS and LAS at the screening of stroke, and the imaging technology should be used for checking the BSC profiles of the patients at the screening for stroke. These insights could be leveraged to develop risk stratification models, especially in individuals with vascular comorbidities (e.g., hypertension and diabetes). Also, this study indicated the necessity of interventions to preserve or enhance WM integrity (e.g., blood pressure control, lifestyle modification, or neuroprotective agents) in order to reduce the risk for stroke in genetically susceptible individuals. The following directions may be considered in corollary studies: validation in longitudinal imaging-genetics cohorts; studies across diverse populations to assess generalizability and subgroup differences; and functional and experimental studies integrating BSC, cerebral perfusion, and metabolic data to clarify underlying mechanisms.

### Limitations

However, there were some limitations to the current study. First, a relaxed threshold (*p* < 5 × 10^−6^) was used to select IVs due to the limited number of genome-wide significant SNPs available for many BSC traits. While this approach increased statistical power, the risk of including weak or pleiotropic instruments was also raised. To mitigate this concern, all instruments had an *F* statistic > 10, and the directionality of associations was verified using the Steiger test. Nevertheless, the relaxed threshold remained a limitation that warrants cautious interpretation. Second, to minimize the effect of ethnic variability, only GWAS data from individuals of European descent were used for the MR analysis. Therefore, the generality of the findings to other populations deserves further exploration and verification, particularly given known ethnic differences in stroke risk and brain connectivity patterns. Third, the accuracy of MR estimates depended in part on the sample size. Therefore, expanding the sample size of other populations is necessary to confirm the reliability of the results. Fourth, some associations showed relatively small odds ratios and wide confidence intervals, which may have limited biological interpretability despite statistical significance. These findings should be considered exploratory and require validation in larger, multiethnic cohorts and experimental models. Finally, uncorrected *p* values were reported with corrected values to improve transparency, but nominally significant findings should be viewed as hypothesis-generating. While the MR analysis provided valuable insights into etiology, these findings should be validated with rigorous RCTs and basic studies before being utilized in the clinic.

### Conclusion

This study found a close association between different BSCs and different subtypes of stroke from a perspective of genetics. While reverse MR analysis suggests a possible bidirectional relationship in some cases, the results warrant further functional validation. These findings provide some valuable insights for early screening and early intervention strategies of stroke.

## Synthesis

Reviewing Editor: Deanna Smith, University of South Carolina Decisions are customarily a result of the Reviewing Editor and the peer reviewers coming together and discussing their recommendations until a consensus is reached. When revisions are invited, a fact-based synthesis statement explaining their decision and outlining what is needed to prepare a revision will be listed below. The following reviewer(s) agreed to reveal their identity: NONE.

The data in this study appear to be sound and potentially useful for clinicians. However, the study is difficult for a non-clinician to understand, and it is suggested that the authors go over the text with that in mind. How would this information actually be used in the clinic? Also, while the study employs robust statistical methods, certain weaknesses highlight the need for cautious interpretation and further validation. For example, some associations showed relatively small odds ratios and wide confidence intervals, which may limit biological interpretability despite statistical significance- functional validation of the identified causal SNPs and biological pathways is lacking. These findings should be considered exploratory and require validation in larger cohorts. Nominally significant findings should be viewed as hypothesis-generating rather than definitive. This should be emphasized throughout the manuscript.
